# The Fractalkine‐CX3CR1 Axis Regulates Non‐inflammatory Osteoclastogenesis by Enhancing Precursor Cell Survival

**DOI:** 10.1002/jbm4.10680

**Published:** 2022-09-22

**Authors:** Yoshikazu Kuboi, Yukiko Kuroda, Masayoshi Ohkuro, Sotaro Motoi, Yoshiya Tomimori, Hisataka Yasuda, Nobuyuki Yasuda, Toshio Imai, Koichi Matsuo

**Affiliations:** ^1^ KAN Research Institute Inc. Kobe Japan; ^2^ Tsukuba Research Laboratories, Eisai Co. Ltd. Tsukuba Japan; ^3^ Laboratory of Cell and Tissue Biology Keio University School of Medicine Tokyo Japan; ^4^ Nagahama Institute for Biochemical Science Oriental Yeast Co. Ltd. Nagahama Japan; ^5^ Bioindustry Division Oriental Yeast Co. Ltd. Tokyo Japan; ^6^ Laboratory of Advanced Therapeutic Target Discovery Kobe University Graduate School of Medicine Kobe Japan

**Keywords:** APOPTOSIS, CX3CR1, FRACTALKINE, OSTEOCLAST PRECURSOR, RANKL

## Abstract

The chemokine fractalkine (FKN) is produced by various cell types, including osteoblasts and endothelial cells in bone tissue, and signals through a sole receptor, CX3CR1, which is expressed on monocytes/macrophages, including osteoclast precursors (OCPs). However, the direct effects of FKN signaling on osteoclast lineage cells under homeostatic noninflammatory conditions remain unclear. Here, we report that FKN regulates mouse OCP survival and primes OCPs for subsequent osteoclast differentiation. Wild‐type but not CX3CR1‐deficient OCPs grown on immobilized FKN showed enhanced osteoclast formation following receptor activator of NF‐κB ligand (RANKL) stimulation, with increased expression of osteoclast differentiation markers. Interestingly, the growth of OCPs on immobilized FKN increased the expression of *Cx3cr1* and *Tnfrsf11a* (*Rank*) transcripts, but following RANKL stimulation, OCPs rapidly downregulated *Cx3cr1* expression. Consistently, anti‐FKN monoclonal antibody (mAb) treatment attenuated RANKL‐induced osteoclast formation on immobilized FKN before, but not during, RANKL stimulation. CX3CR1 and RANK proteins were highly expressed on bone marrow‐derived CD11b^high^ CD115^+^ OCPs. Growth on immobilized FKN prior to RANKL stimulation also increased CD11b^high^ CD115^+^ OCP number and their survival and differentiation potential. In a RANKL‐based mouse model of bone loss, anti‐FKN mAb pretreatment significantly inhibited RANKL‐dependent bone loss. Thus, blocking the FKN‐CX3CR1 axis could represent a therapeutic option in noninflammatory bone loss diseases. © 2022 The Authors. *JBMR Plus* published by Wiley Periodicals LLC on behalf of American Society for Bone and Mineral Research.

## Graphical abstract

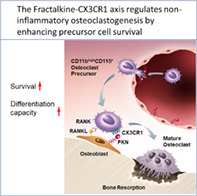

## 1 Introduction

Bone is continuously remodeled through a balance between bone formation by osteoblasts and bone resorption by osteoclasts.^(^ ^1^, ^2^, ^3^ ^)^ Various inflammatory diseases are associated with bone loss,^(^ ^4^ ^)^ whereas excess bone resorption by osteoclasts also occurs under noninflammatory conditions that lead to osteoporosis, most commonly in women after 55 and men after 65 years of age.^(^ ^5^ ^)^ Osteoclasts are tartrate‐resistant acid phosphatase‐positive (TRACP^+^) multinucleated cells generated from osteoclast precursors (OCPs) in the monocyte/macrophage lineage. Their formation requires OCP recruitment to bone tissues and subsequent proliferation, survival, and differentiation, a process that includes cell–cell fusion and polarization.^(^ ^6^ ^)^ OCPs express surface receptors such as the receptor tyrosine kinase CD115 (c‐Fms) and receptor activator of NF‐κB (RANK) (CD265; encoded by *Tnfrsf11a*). Their respective ligands, macrophage‐colony stimulating factor (M‐CSF) and RANK ligand (RANKL), are crucial for osteoclast formation.

Chemokines are structurally related cytokines that regulate the migration and activation of leukocytes and other cell types that express specific seven‐transmembrane‐domain G protein‐coupled receptors. Depending on the arrangement of conserved N‐terminal cysteine residues, chemokines are classified into CXC, CC, C, or CX3C subfamilies.^(^ ^7^ ^)^ Various chemokines and chemokine receptors are critical for bone homeostasis and osteoclastogenesis.^(^ ^8^ ^)^ In particular, CCR1‐deficient mice exhibit osteopenia due to impaired osteoclast and osteoblast function.^(^ ^9^ ^)^ CCR2 is expressed in the osteoclast lineage, and CCR2‐deficient mice show osteopetrosis resulting from markedly reduced numbers of mature osteoclasts.^(^ ^10^ ^)^ Moreover, female CCR2‐deficient mice are resistant to ovariectomy‐induced osteoporosis.^(^ ^10^ ^)^

Fractalkine (FKN), also known as CX3CL1, is the sole member of the CX3C chemokine family^(^ ^11^ ^)^ and exists in both membrane‐bound and soluble forms. The former serves as a cell adhesion molecule between FKN‐ and FKN receptor (CX3CR1)‐expressing cells, whereas the soluble form, which is produced by metalloprotease‐induced shedding, has chemoattractant properties. In bone tissues, osteoblasts express FKN, whereas CX3CR1 expression by osteoblasts remains controversial.^(^ ^12^, ^13^ ^)^ CX3CR1 is expressed on several leukocyte populations, such as monocytes/macrophages, OCPs, effector CD4^+^ T cells, killer CD8^+^ T cells, and dendritic cells.^(^ ^12^, ^13^, ^14^, ^15^, ^16^ ^)^ Circulating monocytes are a source of OCPs, and peripheral blood monocytes can efficiently differentiate into mature osteoclasts in the presence of M‐CSF and RANKL.^(^ ^14^, ^17^ ^)^ However, it is reported that in an irradiated mouse model, vascular endothelial cells also express FKN, which promotes osteoclast recruitment and enhances bone resorption.^(^ ^18^ ^)^ CX3CR1‐deficient mice exhibit increased trabecular and cortical thickness and decreased numbers of osteoclasts, attributable in part to reduced RANKL expression by osteoblasts.^(^ ^12^ ^)^ Overall, FKN has been proposed to stimulate migration and adhesion of CX3CR1‐expressing OCPs to sites of osteoclastogenesis. However, little is known about how OCPs are directly regulated by FKN in the absence of osteoblasts or endothelial cells.

In this study, we first evaluated FKN effects on M‐CSF‐dependent hematopoietic cells using both recombinant FKN and RANKL. We then assessed the activity of the FKN‐CX3CR1 axis in a mouse model of RANKL‐induced bone resorption. In vitro we showed that immobilized FKN enhances RANKL‐induced osteoclastogenesis by directly promoting OCP survival, whereas in vivo we show that treatment with an anti‐FKN monoclonal antibody (mAb) antagonizes RANKL‐induced bone loss in model mice. Our data reveal a possible role for the FKN‐CX3CR1 axis in RANKL‐induced bone loss in the absence of apparent inflammation.

## 2 Materials and Methods

### 2.1 Mice

All animal studies were approved by the Animal Ethics Committee at Eisai Co. Ltd./KAN Research Institute Inc. and were conducted in accordance with their Laboratory Animal Welfare guidelines. Male C57BL/6 mice (6 weeks of age) were obtained from Charles River Laboratories (Tokyo, Japan). Mice were reared within a Japan Health Sciences Foundation–accredited animal facility. CX3CR1‐deficient mice used here were generated in house, as described in the supplementary materials and methods section of Hoshino‐Negishi et al.^(^ ^19^ ^)^ CX3CR1‐deficient mice described here did not show overt bone phenotypes, whereas a previously described CX3CR1‐deficient mouse (B6.129P2(Cg)‐*Cx3cr1* ^*tm1Litt/J*^ mice) exhibited subtle bone gain phenotype.^(^ ^12^ ^)^ Mice were group‐housed under controlled conditions at constant temperature (23°C ± 3°C) and humidity (55% ± 5%), a 12‐hour light/12‐hour dark cycle, and ad libitum access to water and standard pellet food.

### 2.2 Anti‐FKN antibodies

The anti‐mouse FKN mAb (clone 5H8‐4), which is a highly specific neutralizing mAb^(^ ^19^, ^20^, ^21^ ^)^ used in this study, was generated by immunizing Armenian hamsters with recombinant mouse FKN (R&D Systems, Minneapolis, MN).^(^ ^19^ ^)^ Isotype‐matched hamster IgG was generated in Armenian hamsters immunized with 2,4‐dinitrophenol, as previously described.^(^ ^19^, ^21^ ^)^ For some analyses, we used rat anti‐mouse FKN mAb (clone 126315) and rat IgG2A isotype control (clone 54447) purchased from R&D Systems and hamster IgG from Jackson ImmunoResearch Inc. (West Grove, PA).

### 2.3 RNA preparation and quantitative RT‐PCR

Total RNA was isolated from cultured OCPs or osteoclasts using an RNeasy Mini Kit (Qiagen, Hilden, Germany) and reverse‐transcribed to cDNA using a PrimeScript RT Reagent Kit (Qiagen), according to the manufacturer's instructions. Gene expression was measured using an Applied Biosystems 7900 Fast Real‐Time PCR System (Applied Biosystems, Foster City, CA) with the TaqMan Gene Expression Assays software program. Also used were PCR primers specific to TRACP (*Acp5*, Mm00475698_m1), cathepsin K (*Ctsk*, Mm00484039_m1), nuclear factor of activated T cells, cytoplasmic, calcineurin dependent 1 (*Nfatc1*, Mm00479445_m1), dendritic cell‐expressed seven transmembrane protein (*Dcstamp*, Mm01219007_m1), PR domain‐containing 1 (*Blimp1*, *Prdm1*, Mm00476128_m1), B cell leukemia/lymphoma 6 (*Bcl6*, Mm00477633_m1), RANK (*Tnfrsf11a*, Mm00437132_m1), and *Cx3cr1* (Mm00436454_m1). mRNA levels were normalized to that of the housekeeping gene hypoxanthine phosphoribosyltransferase (*Hprt*, Mm01545399_m1). PCR products were quantified using the comparative cycle threshold (Ct) method (2 − ΔΔCt).^(^ ^22^ ^)^

### 2.4 In vitro osteoclastogenesis

Mouse bone marrow cells (BMCs) were differentiated into osteoclasts as described.^(^ ^23^ ^)^ Briefly, nonadherent BMCs were seeded into culture plates (4 × 10^4^ cells per 48‐well plate, 1 × 10^5^ cells per 24‐well plate, or 5 × 10^5^ cells per 35‐mm dish) with or without immobilized FKN (see following discussion) and cultured in α‐MEM (Thermo Fisher Scientific, Waltham, MA) with 10% fetal bovine serum (Thermo Fisher Scientific) containing 10 ng/mL human M‐CSF (R&D Systems). After 2 days, adherent cells were judged to represent crude OCPs and further cultured in the presence of 25 ng/mL soluble mouse RANKL (R&D Systems) and 10 ng/mL M‐CSF to generate osteoclasts. At 3–4 days after RANKL addition, cells were fixed with 4% paraformaldehyde and subjected to TRACP staining using an acid phosphatase, leukocyte (TRACP) kit (Sigma‐Aldrich, St Louis, MO). Using a BIOREVO BZ‐9000 microscope (Keyence, Osaka, Japan), four or five image views were captured, and areas and numbers of TRACP^+^ multinucleated cells (≥3 nuclei) were measured and counted using image analysis software NIS‐Elements (Nikon, Tokyo, Japan) or AxioVision (Zeiss, Oberkochen, Germany). For FKN immobilization, each culture plate was coated with 100 μL recombinant mouse full‐length FKN (5 nM) (R&D Systems) in PBS at 4°C overnight. After extensive PBS washing, nonadherent BMCs were added to the plates. For total cell counts, crude OCPs were fixed in 4% paraformaldehyde after 2 days and stained with Hoechst 33342 solution to detect nuclei (Thermo Fisher Scientific). Using a BIOREVO BZ‐9000 microscope (Keyence), four or five image views were captured, and Hoechst‐positive cells were counted using AxioVision image analysis software (Zeiss).

### 2.5 Flow cytometry

Crude OCPs, BMCs, or peripheral blood leukocytes were stained with antibodies against cell surface markers. Fluorescent‐conjugated or nonconjugated primary antibodies used in this study were as follows: FITC‐, APC‐Cy7‐, or APC‐eFlour 780‐rat anti‐mouse CD11b mAb (M1/70) (eBioscience, San Diego, CA); Alexa Fluor 488, R‐PE‐ or APC‐rat anti‐mouse CD115 mAb (ASF98) (BioLegend, San Diego, CA); APC‐ or APC‐Cy7‐rat Ly6C (AL‐21) (BD Bioscience, San Jose, CA); rat anti‐mouse RANK mAb (R12‐31) (BioLegend or LOB14‐8; Bio‐Rad, Oxford, UK); and R‐PE‐Armenian hamster anti‐mouse CX3CR1 mAb (L2D11).^(^ ^19^ ^)^ Alexa Fluor 647‐conjugated anti‐rat IgG (16‐10A1) (Jackson ImmunoResearch Inc.) was used to detect nonconjugated antibodies. To detect apoptotic cells, we used a fluorescein isothiocyanate (FITC) Annexin V Apoptosis Detection Kit I (BD Bioscience) after cell surface marker staining. All samples were incubated with 1% mouse serum or Mouse BD Fc Block (2.4G2) (BD Bioscience) before antibody staining to prevent nonspecific binding. Data were acquired on a BD FACSCanto II or BD LSRFortessa X‐20 Flow Cytometer (BD Bioscience), and cell sorting was performed on a BD FACSAria II Cell Sorter (BD Bioscience). Acquired data were analyzed using FlowJo software version 10.2 (Tree Star, Ashland, OR). Nonviable cells were excluded based on forward and side scatter profiles and 7‐amino‐actinomycin D (7‐AAD) positivity.

### 2.6 In vivo analysis of RANKL‐induced bone loss following anti‐FKN mAb treatment

Bone loss was evaluated in vivo after a 7‐day pretreatment period. In brief, at 7, 5, 3, 1, and 0 days prior to RANKL injection, mice were administered a single intraperitoneal injection of anti‐FKN mAb (clone 5H8‐4, 400 μg/mouse), control IgG (400 μg/mouse), etanercept (Enbrel; 400 μg/mouse; Pfizer Inc., New York, NY), or PBS. Then, to induce bone loss, on the day after the last mAb treatment, mice in each group were intraperitoneally injected with recombinant human soluble RANKL (0.75 mg/kg) (Oriental Yeast Co. Ltd., Tokyo, Japan), followed by a second RANKL injection on the following day.^(^ ^24^, ^25^ ^)^ For bone histomorphometry and micro‐CT, hindlimbs were isolated 4 hours after the first RANKL injection and 24 hours after the second, respectively. For flow cytometry, peripheral blood and hindlimbs were collected before the first RANKL injection but after anti‐FKN mAb (5H‐8) or control IgG pretreatment described previously, and blood leukocytes and BMCs were stained with antibodies described in the Flow Cytometry section.

### 2.7 Micro‐CT

Micro‐CT of the distal femur was performed using an R_mCT2 scanner (Rigaku, Tokyo, Japan) operated at 90 kV, 160 μA, and 512 projections/360°. Three‐dimensional data were analyzed using Tri/3D‐BON (Ratoc System Engineering, Tokyo, Japan) and ImageJ 1.48 (National Institutes of Health, Bethesda, MD) software. Investigators were blinded during micro‐CT and subsequent analysis.

### 2.8 Statistical analysis

Statistical significance was determined using GraphPad Prism software version 7.02 (GraphPad Software, La Jolla, CA). Data were analyzed using a two‐tailed Student's *t*‐test or by one‐way ANOVA followed by Dunnett's multiple comparisons test. A value of *p* < 0.05 was considered statistically significant. All data points are plotted.

## 3 Results

### 3.1 BMCs grown on immobilized FKN exhibit enhanced expression of osteoclast differentiation markers

**Fig. 1:**
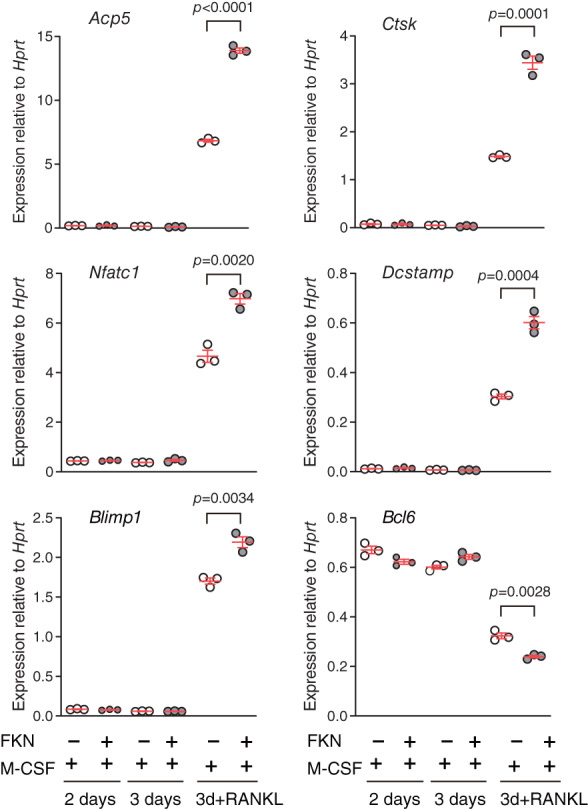
Bone marrow cells (BMCs) cultured on immobilized fractalkine (FKN) show enhanced expression of osteoclast differentiation markers following receptor activator of NF‐κB ligand (RANKL) induction. Expression levels of osteoclast differentiation markers were measured by qRT‐PCR and normalized to that of *Hprt*. BMCs were cultured for 2 days with macrophage‐colony stimulating factor (M‐CSF) in the presence or absence of immobilized FKN, followed by treatment with M‐CSF only or M‐CSF plus RANKL for an additional 24 hours (3 days and 3d + RANKL, respectively). Data are presented as means ± SEM, unpaired *t*‐test.

To assess FKN effects on OCPs and/or osteoclasts, we monitored expression of transcripts encoding osteoclast differentiation markers (*Acp5*, *Ctsk*, *Nfatc1*, *Dcstamp*, and *Blimp1*) by culturing nonadherent BMCs on plates coated without or with immobilized recombinant FKN, as a means to mimic membrane‐bound FKN. After 2 days of culture with M‐CSF in the presence or absence of immobilized FKN, expression of osteoclast differentiation markers remained low (Fig. 1, 2 days), a pattern that remained unchanged after an additional 24 hours, irrespective of the presence of FKN (Fig. 1, 3 days). By contrast, when we included RANKL with M‐CSF for the last 24 hours, *Acp5*, *Ctsk*, *Nfatc1*, *Dcstamp*, and *Blimp1* expression increased, while expression of *Bcl6*, a negative regulator of osteoclast differentiation,^(^ ^26^ ^)^ decreased. All effects were significantly greater in cells grown on immobilized FKN than in control cells grown without FKN (Fig. 1, 3d+RANKL), suggesting that immobilized FKN potentiates RANKL‐induced osteoclast differentiation.

**Fig. 2:**
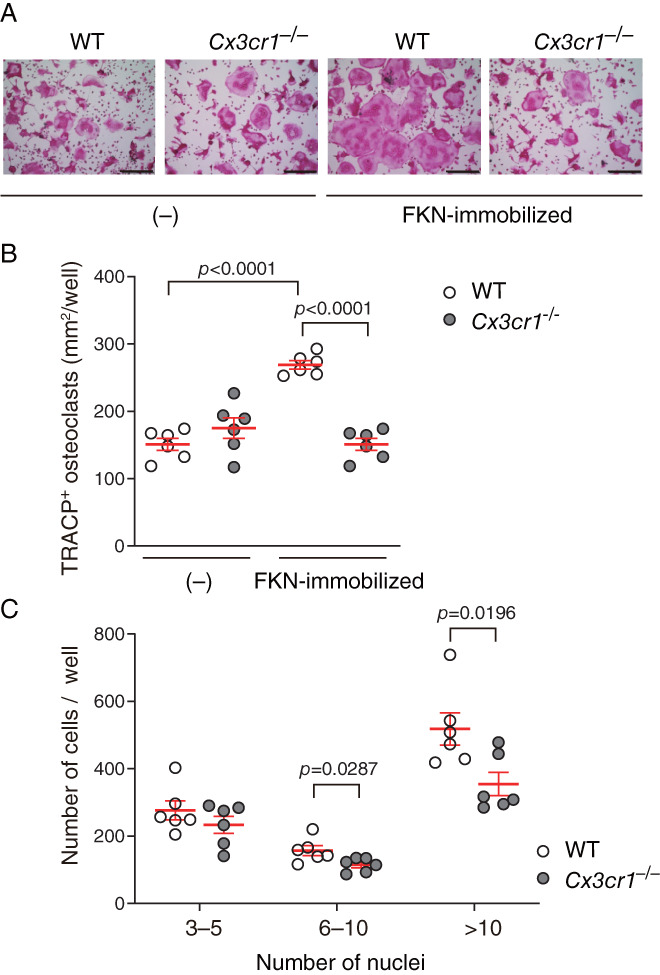
CX3CR1 deficiency blocks fractalkine (FKN)‐enhanced osteoclast formation following receptor activator of NF‐κB ligand (RANKL) induction. Bone marrow cells (BMCs) were isolated from wild‐type or CX3CR1‐deficient mice and differentiated in the presence of macrophage‐colony stimulating factor (M‐CSF) and RANKL, with or without immobilized FKN. (*A*) Representative images of cells visualized using a tartrate‐resistant acid phosphatase‐positive (TRACP) activity stain. Scale bars, 300 μm. (*B*) TRACP^+^ areas corresponding to mononucleated and multinucleated cells. Data are shown as means ± SEM, Dunnett's multiple comparisons test. (*C*) Numbers of TRACP^+^ multinucleated cells derived from wild‐type or CX3CR1‐deficient mice. Cells containing the indicated number of nuclei were counted. Data are shown as means ± SEM, unpaired *t*‐test.

**Fig. 3:**
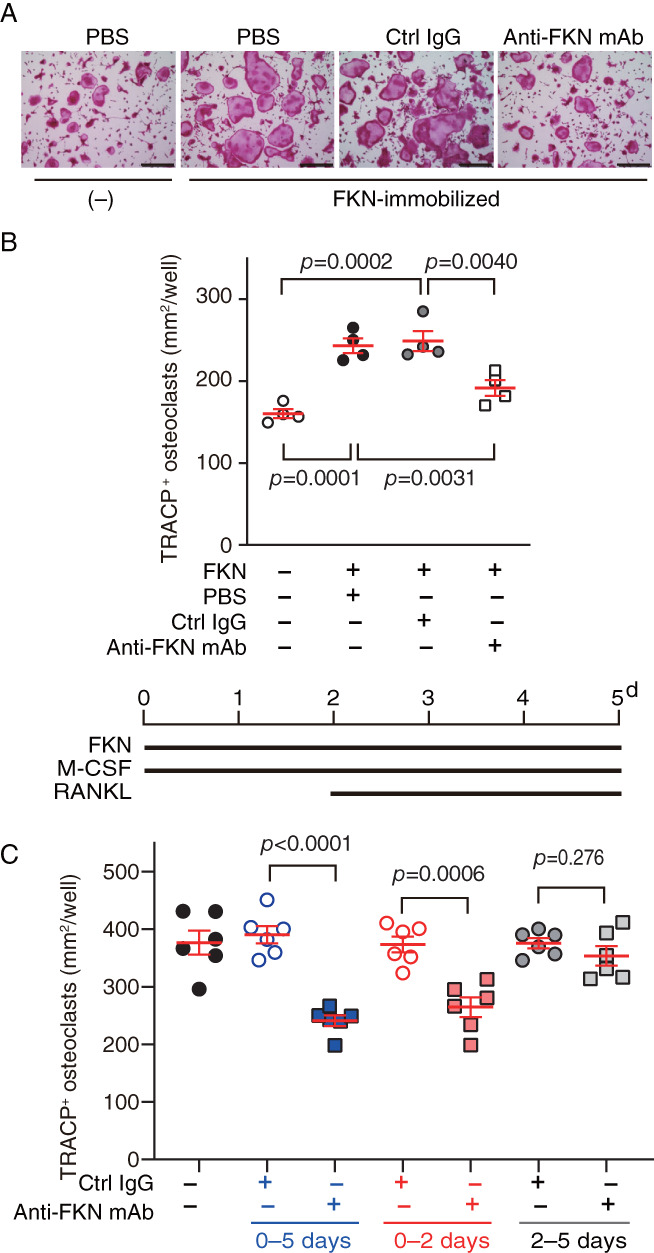
Anti‐fractalkine (FKN) monoclonal antibody (mAb) antagonizes FKN‐mediated enhancement of osteoclast formation. (*A*) Representative images of cells visualized by a tartrate‐resistant acid phosphatase (TRACP) activity stain after indicated treatments. Scale bars, 300 μm. Ctrl, control. Anti‐FKN mAb (clone 126315). (*B*) (upper panel) TRACP^+^ areas corresponding to mononucleated and multinucleated cells in indicated treatment conditions. Anti‐FKN mAb (clone 126315), control (ctrl) IgG, or PBS was added on Day 0. Data are presented as means ± SEM, Dunnett's multiple comparisons test. (Lower panel) schematic illustrating timeline for treating bone marrow cells (BMCs) with FKN, macrophage‐colony stimulating factor (M‐CSF), and receptor activator of NF‐κB ligand (RANKL). (*C*) Osteoclasts were induced from BMCs using M‐CSF and RANKL in the presence of ctrl IgG or anti‐FKN mAb (clone 5H8‐4) in FKN‐immobilized conditions. Data are presented as means ± SEM, unpaired *t*‐test.

### 3.2 CX3CR1 deficiency blocks FKN effects on osteoclastogenesis

We next evaluated effects of CX3CR1 signaling on RANKL‐induced osteoclast differentiation in BMCs grown on immobilized FKN. To do so, we cultured nonadherent BMCs derived from wild‐type or CX3CR1‐deficient mice for 2 days in the presence of M‐CSF alone, followed by stimulation with M‐CSF plus RANKL for 3 days, and then measured the area and number of TRACP^+^ multinucleated cells containing ≥3 nuclei (Fig. 2*A*). Areas of TRACP^+^ cells on immobilized FKN were significantly greater in cultures of wild‐type relative to CX3CR1‐deficient BMCs (*Fig*. *2B*). In cells cultured on immobilized FKN, the numbers of relatively small TRACP^+^ cells containing 3–5 nuclei were comparable between wild‐type and CX3CR1‐deficient cells, while the number of larger TRACP^+^ cells containing 6–10 or >10 nuclei significantly decreased in CX3CR1‐deficient compared with wild‐type cells (Fig. 2*C*). These results suggest that enhanced induction of osteoclast formation following RANKL stimulation seen in the presence of immobilized FKN requires CX3CR1, although cultures of CX3CR1‐deficient BMCs are also likely to have contained fewer OCPs at the start of culture due to their reduced survival (Fig. 5).

### 3.3 Treatment of BMCs with anti‐FKN mAb prior to RANKL stimulation blocks FKN enhancement of osteoclast differentiation

Our findings suggest that BMCs cultured on immobilized FKN show enhanced osteoclast differentiation following RANKL stimulation. Thus, we asked whether culture in soluble rather than on immobilized FKN would have comparable effects. To answer this question, we grew nonadherent BMCs for 2 days on uncoated plates in media containing 25 ng/mL soluble FKN and then added RANKL to culture media for 3 more days. Based on the area of resultant TRACP^+^ cells, osteoclast differentiation following RANKL treatment was equivalent in the presence or absence of soluble FKN (Fig. S1), suggesting that growth on immobilized FKN rather than in media containing soluble FKN induces signaling required to enhance osteoclast differentiation following RANKL stimulation.

Next, to determine FKN specificity, we treated BMCs growing on immobilized FKN with anti‐FKN mAb at Day 0 of in vitro culture. mAb treatment for 5 days till the end of osteoclastogeneis significantly blocked enhanced osteoclast differentiation seen in the absence of antibody based on TRACP activity staining (Fig. 3*A*) and TRACP^+^ cell counts (Fig. 3*B*).

To determine the time window during which anti‐FKN mAb was effective during RANKL‐induced osteoclast differentiation, we added anti‐FKN mAb to cultures for either the entire 5‐day differentiation period (0–5 days), only for the first 2 days (0–2 days), or only for the last 3 days (2–5 days), the period when RANKL was added to cultures. As shown in Fig. 3*C*, anti‐FKN mAb treatment for the entire 5‐day period inhibited osteoclast differentiation by RANKL. Comparable treatment in the early (0–2 days) period also had a significant inhibitory effect; by contrast, we observed no inhibitory effects when anti‐FKN mAb was added together with RANKL (2–5 days) (Fig. 3*C*). Overall, these findings suggest that BMC interaction with membrane‐bound FKN does not directly block RANK signaling but rather alters a cell's ability to respond to RANKL treatment.

### 3.4 Crude OCPs grown on immobilized FKN show enhanced RANK and CX3CR1 expression

**Fig. 4:**
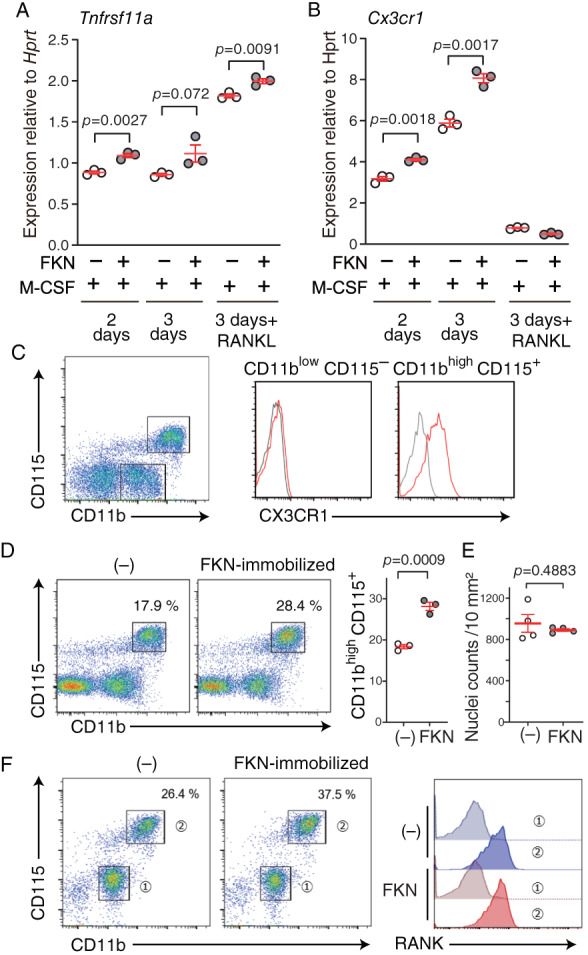
Effects of immobilized fractalkine (FKN) on CD11b^high^ CD115^+^ osteoclast precursors (OCPs) expressing RANK (*Tnfrsf11a*) and CX3CR1 (*Cx3cr1*). (*A*) *Tnfrsf11a* and (*B*) *Cx3cr1* expression normalized to that of *Hprt* was measured by qRT‐PCR. Nonadherent bone marrow cells (BMCs) were cultured with macrophage‐colony stimulating factor (M‐CSF) for 2 or 3 days in the presence or absence of immobilized FKN. “+RANKL” indicates RANKL treatment for the last 24 hours. Data are presented as means ± SEM, unpaired *t*‐test. (*C*) Flow cytometry analysis of CX3CR1 expression on crude OCPs. BMCs were cultured 2 days in M‐CSF in the presence or absence of immobilized FKN and stained for CD11b and CD115 (*n* = 3). Gated CD11b^low^ CD115^−^ and CD11b^high^ CD115^+^ populations are indicated in the left panel. Right panels show CX3CR1 fractions gated for CD11b^low^ CD115^−^ or CD11b^high^ CD115^+^. Gray and red lines indicate cells without and with immobilized FKN, respectively. (*D*) Flow cytometry analysis of crude OCPs for CD11b^high^ CD115^+^ population. Nonadherent BMCs were cultured for 2 days with M‐CSF without or with immobilized FKN and analyzed by flow cytometry. Graph on right shows the percentage of CD11b^high^ CD115^+^ cells in each condition. Data are presented as means ± SEM, unpaired *t*‐test. (*E*) Nuclei counts of crude OCPs, based on Hoechst staining in 48‐well plates, prior to flow cytometry analysis. (*F*) Flow cytometry analysis of crude OCPs for CD11b^low^ CD115^−^ (Gate 1) or CD11b^high^ CD115^+^ (Gate 2) populations. Nonadherent BMCs were cultured for 2 days with M‐CSF without or with immobilized FKN and analyzed by flow cytometry. RANK expression for each gate is shown at right.

To assess whether FKN regulates RANK and CX3CR1 expression, we measured respective *Tnfrsf11a* and *Cx3cr1* transcript levels during osteoclastogenesis in cells grown on immobilized FKN. Prior to RANKL stimulation, *Tnfrsf11a* expression was significantly higher in crude OCPs grown 2 days on immobilized FKN relative to cells grown without FKN (Fig. 4*A*). *Tnfrsf11a* levels in both groups were further elevated after 24 hours of RANKL stimulation, especially in cells grown on immobilized FKN (Fig. 4*A*). Also, prior to RANKL addition, *Cx3cr1* expression was higher in crude OCPs grown on immobilized FKN relative to cells grown without FKN (Fig. 4*B*). Strikingly, after 24 hours of RANKL stimulation, *Cx3cr1* expression was robustly downregulated in cells grown with and without FKN (Fig.  4*B*, 3 days +RANKL). These data indicate that FKN‐CX3CR1 signaling occurs before RANKL addition and is downregulated once cells become differentiated. Moreover, these data suggest that the period in which anti‐FKN mAb can suppress FKN enhancement of osteoclastogenesis correlates with the period of *Cx3cr1* expression.

### 3.5 The CD11b^high^ CD115 ^+^ OCP population increases in cells grown on immobilized FKN

We next used flow cytometry to determine which cell populations expressed RANK and CX3CR1 among crude OCPs, namely, adherent BMCs obtained after M‐CSF‐induced differentiation for 2 days of nonadherence. That analysis revealed three distinct subpopulations: CD11b^−^ CD115^−^, CD11b^low^ CD115^−^, and CD11b^high^ CD115^+^ (Fig. 4*C*). CD11b^high^ CD115^+^ cells, but not CD11b^low^ CD115^−^ or CD11b^−^ CD115^−^ cells, expressed CX3CR1 without and with immobilized FKN (Fig. 4*C* and Fig. S2). Consistent with transcript analysis, RANKL stimulation for 1 day followed by flow cytometry analysis revealed that cell surface CX3CR1 expression in the CD11b^high^ CD115^+^ population decreased in the presence or absence of immobilized FKN (Fig. S3). Furthermore, the percentage of cells in the CD11b^high^ CD115^+^ population significantly increased over the 2 days when cells were grown on immobilized FKN prior to flow cytometry (Fig. 4*D*). That percentage reflected the absolute number of cells, since total nuclei counts of crude OCPs used for flow cytometry analysis were comparable between cells with and without immobilized FKN treatment (Fig. 4*E*). Importantly, RANK expression was similarly detectable in CD11b^high^ CD115^+^ cell populations with or without immobilized FKN (Fig. 4*F*). These data suggest that growth on immobilized FKN increases the number of CD11b^high^ CD115^+^ OCPs that express RANK.

### 3.6 Immobilized FKN promotes survival and differentiation capacity of CD11b^high^ CD115 ^+^ OCPs

**Fig. 5:**
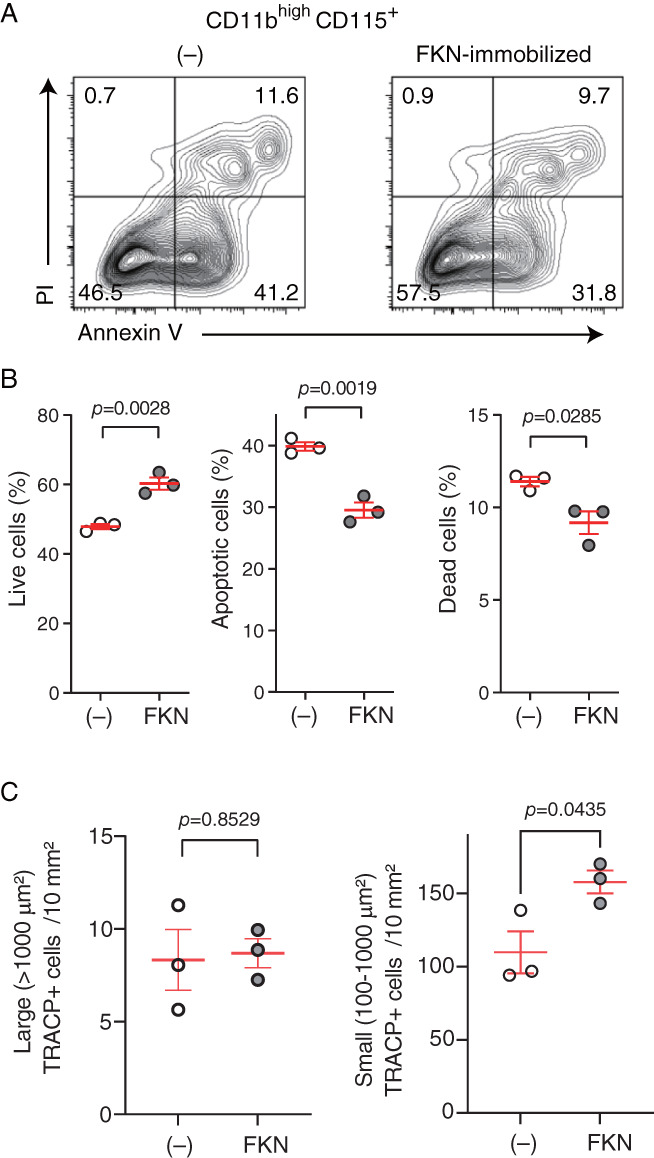
Growth on immobilized fractalkine (FKN) enhances CD11b^high^ CD115^+^ osteoclast precursor (OCP) survival and differentiation capacity. (*A*) Flow cytometry of bone marrow cells (BMCs) cultured for 2 days in the presence of macrophage‐colony stimulating factor (M‐CSF) with or without immobilized FKN and gated for CD11b^high^ CD115^+^ OCPs. Apoptotic and dead CD11b^high^ CD115^+^ OCPs were assessed by Annexin V and PI staining. Numbers indicate percentage of cells in each quadrant. Representative data plots are shown. (*B*) Quantification of living, apoptotic, or dead cells from analysis shown in (*A*) (*n* = 3). (*C*) Number of large (>1000 μm^2^) or small (100–1000 μm^2^) tartrate‐resistant acid phosphatase‐positive (TRACP^+^) cells per 10 mm^2^. Sorted CD11b^high^ CD115^+^ were cultured for 2 days with M‐CSF without or with immobilized FKN. TRACP^+^ cells were counted in three wells of a 48‐well plate for each treatment. Data are presented as means ± SEM, unpaired *t*‐test.

To determine the mechanisms underlying the increase in OCP number seen in the presence of immobilized FKN, we used flow cytometry to analyze apoptosis based on Annexin V and propidium iodide (PI) staining in the OCP population when cells were grown in the presence or absence of immobilized FKN (Fig. 5*A*). Compared to CD11b^high^ CD115^+^ OCPs not grown on FKN, CD11b^high^ CD115^+^ OCPs grown on immobilized FKN exhibited significantly higher numbers of viable (Annexin V^−^ and PI^−^) cells and smaller fractions of early (Annexin V^+^ and PI^−^) or late (or dead; Annexin V^+^ and PI^+^) apoptotic cells (Fig. 5*B*), suggesting that growth on immobilized FKN enhances their survival.

Next, we sorted equal numbers of CD11b^high^ CD115^+^ OCPs that had been cultured with or without immobilized FKN and showed comparable viability and determined their differentiation capacity. As shown in Fig. 5*C*, although the number of large TRACP^+^ cells (>1000 μm^2^) was comparable in both groups, the number of small TRACP^+^ cells with a diameter of 100–1000 μm^2^ significantly increased in cells grown on FKN, suggesting that FKN signaling through CX3CR1 promotes OCP survival and differentiation capacity.

### 3.7 Anti‐FKN mAb treatment antagonizes RANKL‐induced bone loss in model mice

**Fig. 6:**
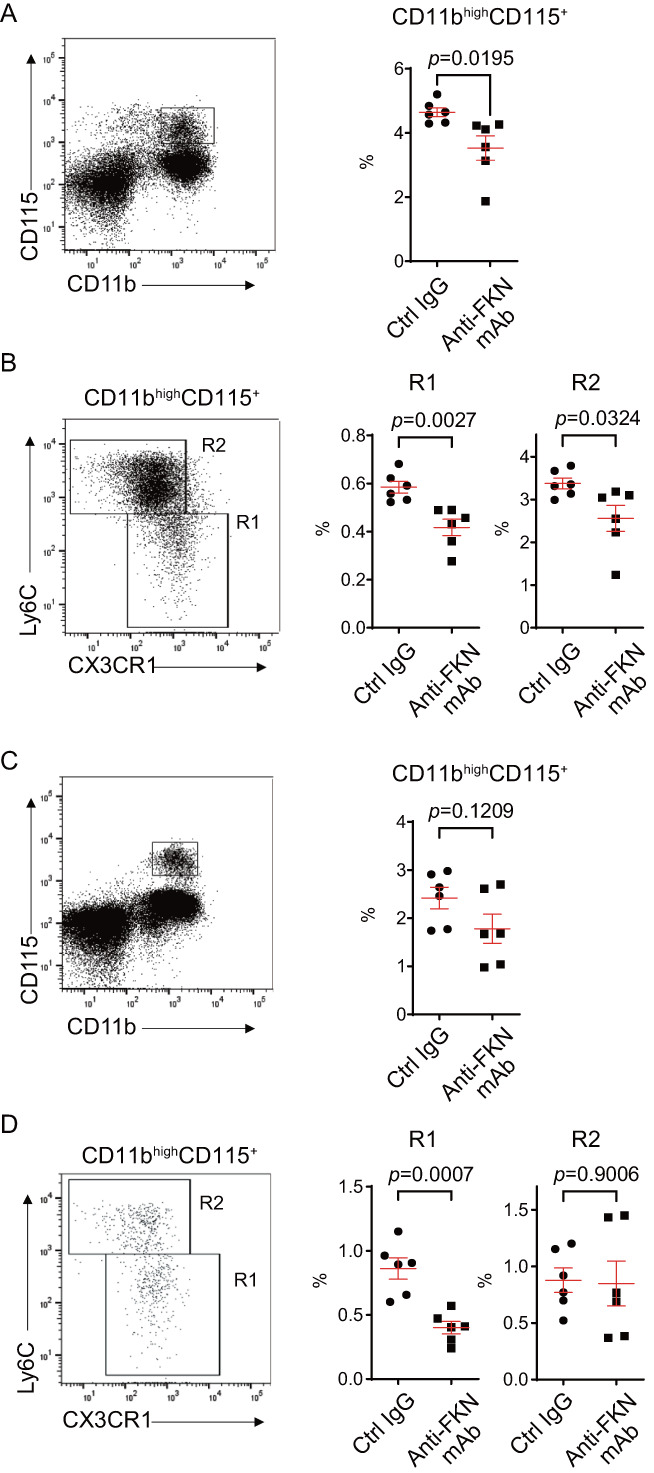
Anti‐fractalkine (FKN) monoclonal antibody (mAb) treatment decreases the number of CD11b^high^ CD115^+^ Ly6C^low^ CX3CR1^high^ cells in bone marrow and peripheral blood in vivo. Bone marrow cells (BMCs) and blood leukocytes isolated from mice pretreated for 7 days with control IgG or anti‐FKN mAb (clone 5H8‐4) were stained with cell surface markers (CD11b, CD115, Ly6C, and CX3CR1) and analyzed by flow cytometry. (*A*) Representative fluorescence‐activated cell sorting (FACS) dot plots from BMCs and percentage of CD11b^high^ CD115^+^ OCPs. (*B*) R1 and R2 subpopulations from each pretreatment. (*C*) Representative FACS dot plots from blood leukocytes and percentage of CD11b^high^ CD115^+^ osteoclast precursors (OCPs). (*D*) R1 and R2 subpopulations from each pretreatment. Means ± SEM, *n* = 6 mice per pretreatment.

Finally, we investigated the effects of anti‐FKN mAb treatment in vivo in a RANKL‐induced bone loss mouse model. To create this model, we intraperitoneally injected mice with anti‐FKN mAb (clone 5H8‐4), control IgG, or PBS once on Days 7, 5, 3, 1, and 0 before intraperitoneal RANKL injection, also administered on Day 0. Mice were euthanized 4 hours after RANKL injection, and their femurs were analyzed by bone histomorphometry to assess osteoclast parameters at the acute phase of RANKL‐induced bone loss. Differences among the three treatment groups were not statistically significant (Fig. S4); therefore, we analyzed the CD11b^high^ CD115^+^ OCP population in bone marrow and peripheral blood prior to RANKL injection to monitor possible effects of anti‐FKN mAb on OCPs. In bone marrow, the percentage of CD11b^high^ CD115^+^ OCPs significantly decreased following in vivo anti‐FKN mAb pretreatment (Fig. 6*A*). CD11b^high^ CD115^+^ cells in BMCs were further analyzed for Ly6C and CX3CR1 expression, which had been used for OCP analysis.^(^ ^27^, ^28^ ^)^ The percentage of both Ly6C^low^ CX3CR1^high^ (R1) and Ly6C^high^ CX3CR1^low^ (R2) subpopulations also significantly decreased after anti‐FKN mAb pretreatment (Fig. 6*B*). In peripheral blood, suppression of CD11b^high^ CD115^+^ OCPs by anti‐FKN mAb pretreatment was minimal (Fig. 6*C*). Further analysis revealed that the percentage of Ly6C^low^ CX3CR1^high^ (R1) subpopulation but not that of the Ly6C^high^CX3CR1^low^ (R2) population in blood leukocytes significantly decreased after anti‐FKN mAb treatment (Fig. 6*D*).

**Fig. 7:**
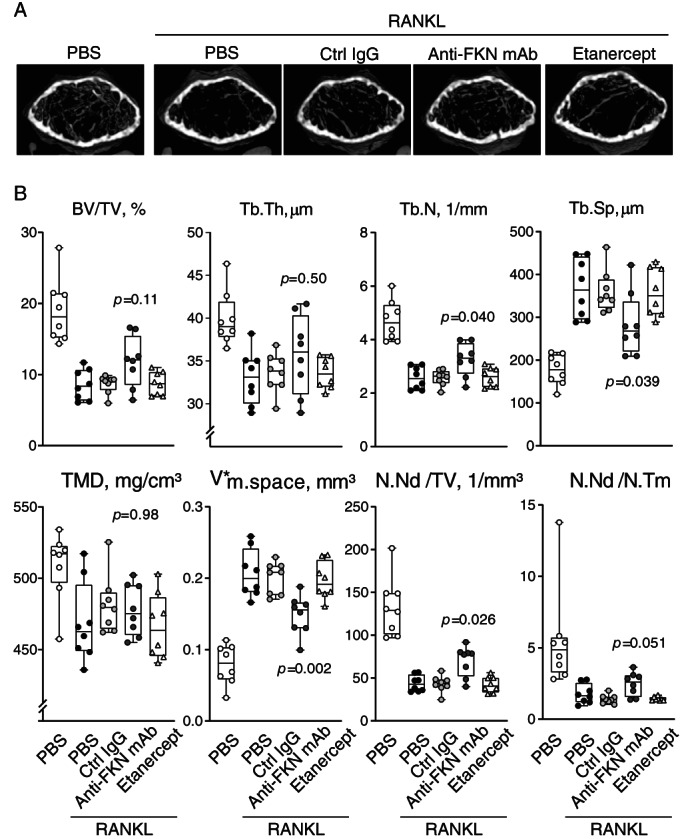
Anti‐fractalkine (FKN) monoclonal antibody (mAb) treatment suppresses receptor activator of NF‐κB ligand (RANKL)‐induced bone loss in vivo. (*A*) Micro‐CT images of femurs isolated from control PBS‐treated mice or RANKL‐treated mice in a RANKL‐dependent mouse model of bone loss. As indicated, only RANKL‐treated mice were also pretreated with PBS, control IgG, anti‐FKN mAb (clone 5H8‐4), or etanercept. Representative images are shown (*n* = 8 per group) and quantification is provided in (*B*). Scale bar, 500 μm. (*B*) Bone morphometric parameters in control mice not administered RANKL and in indicated groups of RANKL‐treated mice (*n* = 8 per group). Parameters evaluated are shown at top of graphs. Boxplot indicates median, interquartile range, maximum, and minimum. TMD, tissue mineral density; BV/TV, bone volume/tissue volume; Tb.Th, trabecular thickness; Tb.N, trabecular number; Tb.Sp, trabecular separation; V*_m.space_, star volume of marrow space; N.Nd/TV, node number; N.Nd/N.Tm, node number‐to‐terminus number ratio.

To push OCP differentiation toward osteoclasts, we then injected RANKL a second time 1 day after the first RANKL injection and sacrificed animals for analysis 24 hours later. For comparison, we included the tumor necrosis factor (TNF)‐α blocker etanercept as an additional pretreatment group. We then analyzed isolated femurs three‐dimensionally using micro‐CT. Control mice pretreated with PBS or control IgG showed reduced trabecular bone mass following RANKL treatment. In contrast, anti‐FKN mAb pretreatment partially blocked loss of trabecular bone, whereas etanercept treatment did not (Fig. 7*A*). We then quantified several micro‐CT‐based morphometric parameters of trabecular bone,^(^ ^29^, ^30^, ^31^, ^32^ ^)^ including bone volume/tissue volume (BV/TV), trabecular thickness (Tb.Th), trabecular number (Tb.N), trabecular separation (Tb.Sp), tissue mineral density (TMD), star volume of marrow space (V*_m.space_), node number (N.Nd/TV), and node number‐to‐terminus number ratio (N.Nd/N.Tm) (Fig. 7*B*). Anti‐FKN mAb pretreatment significantly prevented decreases in trabecular number and node number relative to other groups. Consistently, increases in trabecular separation and marrow space were significantly blocked by anti‐FKN mAb pretreatment (Fig. 7*B*). These data indicate that anti‐FKN mAb pretreatment modulates OCP states, partially inhibits RANKL‐induced bone loss in vivo, and preserves trabecular bone microarchitecture.

## 4 Discussion

**Fig. 8:**
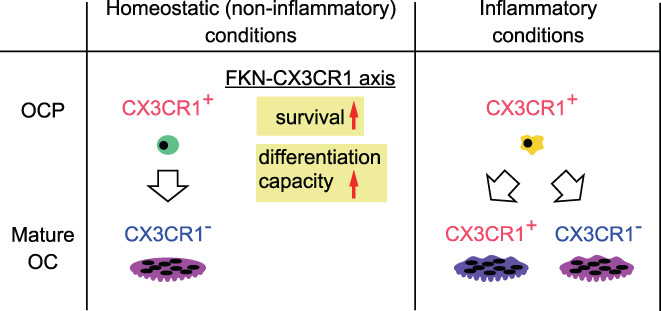
Model showing proposed CX3CR1 expression patterns during osteoclastogenesis under homeostatic or inflammatory conditions. In homeostatic osteoclastogenesis, the fractalkine (FKN)‐CX3CR1 axis functions only in osteoclast precursors (OCPs) because CX3CR1 is not expressed in mature osteoclasts. FKN‐CX3CR1 signaling directly regulates survival and differentiation capacity of homeostatic OCPs and thus promotes osteoclast differentiation.

This study suggests that FKN‐CX3CR1 activity directly regulates the survival and differentiation capacity of homeostatic OCPs in noninflammatory bone loss disease such as osteoporosis (Fig. 8). We also show that *Cx3cr1* transcript levels decrease in noninflammatory mature osteoclasts in the presence of RANKL. Indeed, it was previously reported that RANKL treatment downregulated CX3CR1 in a mouse macrophage line and in CX3CR1‐EGFP mice.^(^ ^12^, ^33^ ^)^ These findings are in sharp contrast to inflammatory OCPs, which continue to express CX3CR1 after osteoclast differentiation^(^ ^33^, ^34^, ^35^ ^)^ (Fig. 8).

Specifically, we report that homeostatic OCPs cultured on immobilized FKN, but not in the presence of soluble FKN, exhibit increased expression of osteoclast differentiation markers following RANKL induction. Our findings suggest that clustering of cell‐surface CX3CR1 on OCPs enhances RANKL‐induced osteoclastogenesis. Notably, it remains unknown whether the receptor CX3CR1 on OCPs are exposed to the same number of soluble and immobilized FKN molecules and whether both forms of FKN are differentially recognized by OCPs. It is also possible that efficiency of CX3CR1 internalization, movement, or multimerization differs in the presence of soluble versus immobilized FKN. Curiously, cognitive impairment seen in CX3CL1‐deficient mice is rescued more effectively by soluble than membrane‐bound FKN.^(^ ^36^ ^)^

We observed that CX3CR1‐deficient OCPs grown on immobilized FKN were refractory to RANKL induction: While they differentiated into osteoclasts, those osteoclasts were smaller and contained fewer nuclei than did wild‐type control cells induced by RANKL. CX3CR1 deficiency decreased not only the number of multinucleated mature osteoclasts but their total surface area. In wild‐type cells, growth on immobilized FKN enhanced expression of osteoclast fusion‐related *Dcstamp* following RANKL induction. Collectively, these data suggest that perturbation of the FKN‐CX3CR1 axis impairs osteoclast fusion.

Importantly, treatment of OCP cultures with anti‐FKN mAb effectively abrogated FKN‐enhanced osteoclast differentiation, but only when antibody was added before RANKL stimulation: When antibody and RANKL were added simultaneously, FKN‐enhanced osteoclastogenesis was unchanged. These findings are consistent with previous observations made in osteoclastogenic co‐cultures of osteoblasts exposed to rat anti‐FKN mAb (clone 126315) throughout the culture period.^(^ ^13^ ^)^ Immobilized FKN also enhanced expression of *Cx3cr1* in crude OCPs following RANKL induction, while, as noted previously, CX3CR1 expression was downregulated in the presence of RANKL. These observations suggest that FKN‐CX3CR1 signaling is most critical for osteoclastogenesis at the OCP stage.

In accordance with a previous study,^(^ ^37^ ^)^ we defined the CD11b^high^ CD115^+^ (c‐Fms^+^) cell population in crude OCPs as enriched in OCPs. Consistently, CD11b^high^ CD115^+^ cells specifically expressed high levels of RANK and CX3CR1. Importantly, the number of cells in the OCP population significantly increased when cells were grown on immobilized FKN. Our data suggest that this increase could be due to the suppression of apoptosis following FKN‐CX3CR1 interaction. Consistent with these findings, CX3CR1 functions in monocyte homeostasis by promoting cell survival,^(^ ^27^ ^)^ and membrane‐anchored FKN is required for monocyte survival in vivo.^(^ ^28^ ^)^ All of these findings strongly suggest that osteoclastogenesis is likely regulated at the level of OCP survival via FKN, which is expressed on stromal cells, including osteoblasts. FKN‐induced OCP survival is likely mediated via AKT and ERK activation, as has been shown for human monocytes.^(^ ^38^ ^)^ Recently, in analysis performed in living mice, physiological oxygen tension in CX3CR1‐expressing monocytes was determined to be 5.3%.^(^ ^39^ ^)^ Since hypoxia increases CX3CR1 expression in some cancer cells,^(^ ^40^ ^)^ OCP survival and differentiation under hypoxic conditions may be regulated by FKN‐CX3CR1 signaling,^(^ ^41^ ^)^ although the mechanisms underlying these outcomes remain unknown.

Our findings suggest that OCPs could serve as therapeutic targets in some bone loss diseases. Specifically, anti‐FKN mAb pretreatment significantly prevented bone loss and altered bone histomorphometry parameters in a RANKL‐induced bone resorption model in mice. Although TNF‐α also serves as a potential therapeutic target for osteoporosis,^(^ ^42^ ^)^ in our in vivo model, etanercept did not prevent bone loss, likely because RANKL‐induced bone loss is noninflammatory. Indeed, RANKL pretreatment can be anti‐inflammatory at least in mice.^(^ ^43^ ^)^ Besides the indirect suppressive effects of FKN‐CX3CR1 activity on osteoclasts via osteoblasts,^(^ ^12^ ^)^ we also show that FKN‐CX3CR1 signaling modulates OCP survival in the context of bone resorption, independently of inflammatory and immunomodulatory activities. Therefore, blocking FKN‐CX3CR1 signaling could serve as a useful therapeutic option not only for rheumatoid arthritis^(^ ^19^ ^)^ but for osteoporosis by specifically regulating noninflammatory OCPs without directly affecting mature osteoclasts.

## AUTHOR CONTRIBUTIONS

**Yoshikazu Kuboi:** Conceptualization; data curation; formal analysis; investigation; methodology; resources; software; validation; visualization; writing – original draft. **Yukiko Kuroda:** Conceptualization; data curation; investigation; methodology; resources; software; visualization; writing – review and editing. **Masayoshi Ohkuro:** Data curation; formal analysis; investigation. **Sotaro Motoi:** Formal analysis; investigation. **Yoshiya Tomimori:** Methodology; resources. **Hisataka Yasuda:** Methodology; resources. **Nobuyuki Yasuda:** Methodology; supervision. **Toshio Imai:** Conceptualization; project administration; supervision; writing – review and editing. **Koichi Matsuo:** Conceptualization; data curation; formal analysis; funding acquisition; investigation; project administration; software; supervision; validation; visualization; writing – review and editing.

## Competing Interests

Yoshikazu Kuboi, Masayoshi Ohkuro, Satoru Motoi, Nobuyuki Yasuda, and Toshio Imai are employees of KAN Research Institute Inc., Japan. Yoshiya Tomimori and Hisataka Yasuda are employees of Oriental Yeast Co. Ltd., Japan. This research was partly supported by a grant from KAN Research Institute Inc. to Koichi Matsuo and Yukiko Kuroda. KAN Research Institute Inc. is a subsidiary of Eisai Co. Ltd.

### PEER REVIEW

The peer review history for this article is available at https://publons.com/publon/10.1002/jbm4.10680.

## Supporting information


**Figure S1.** Effect of soluble fractalkine (FKN) on receptor activator of NF‐κB ligand (RANKL)‐induced osteoclast formation in vitro. (Left) Osteoclasts were differentiated from bone marrow cells (BMCs) over 4 days in the presence of macrophage‐colony stimulating factor (M‐CSF) and RANKL at indicated concentrations of soluble FKN, and areas covered by tartrate‐resistant acid phosphatase‐positive (TRACP^+^) cells were measured. Data are presented as means ± SEM. (Right) Representative images of cells either untreated (upper panel) or treated (lower panel) with 10 nM soluble FKN. Scale bars, 300 μm.
**Figure S2**. Cell surface expression of CX3CR1 on CD11b^−^ CD115^−^ cells 2 days after macrophage‐colony stimulating factor (M‐CSF) stimulation. BMCs were cultured 2 days in the presence of M‐CSF and then analyzed by flow cytometry. (Left) Representative data plot. (Right) CX3CR1 (upper panel) and RANK (lower panel) expression on CD11b^−^ CD115^−^ cells. Gray lines, isotype IgG; red lines, anti‐CX3CR1 mAb or anti‐receptor activator of NF‐κB (RANK) monoclonal antibody.
**Figure S3**. Decreased cell surface expression of CX3CR1 on CD11b^high^ CD115^+^ osteoclast precursors (OCPs) at 1 day after receptor activator of NF‐κB ligand (RANKL) stimulation. Bone marrow cells (BMCs) were cultured 2 days in the presence of macrophage‐colony stimulating factor (M‐CSF) followed by RANKL stimulation for 1 day and subsequent flow cytometry analysis. (Left) Representative data plot. (Right) CX3CR1 fraction gated for CD11b^high^ CD115^+^. Gray and red lines: cells grown without and with immobilized fractalkine, respectively.
**Figure S4**. Bone histomorphometry analysis osteoclast parameters at acute phase of receptor activator of NF‐κB ligand (RANKL)‐induced bone loss. Quantitative analysis of trabecular bone volume per tissue volume (BV/TV), osteoclast number per bone perimeter (OC number/Perim), and osteoclast area per bone perimeter (OC area/Perim) in femoral sections. Femurs were isolated from control PBS‐treated mice (*n* = 4) or RANKL‐treated mice pretreated with PBS, control IgG, or anti‐fractalkine monoclonal antibody (clone 5H8‐4) (*n* = 8 per group). Boxplots indicate median, interquartile range, maximum, and minimum.

## Data Availability

Data supporting this study's findings are available from the corresponding author upon reasonable request.
