# Supplementary material for: The Fractalkine‐CX3CR1 Axis Regulates Non‐inflammatory Osteoclastogenesis by Enhancing Precursor Cell Survival
Source: JBMR Plus. 2022 Sep 22;6(10):e10680. doi: 10.1002/jbm4.10680 (PMC9549724; doi:10.1002/jbm4.10680)
Supplement: Supplementary file 1 — Figure S1. Effect of soluble fractalkine (FKN) on receptor activator of NF‐κB ligand (RANKL)‐induced osteoclast formation in vitro. (Left) Osteoclasts were differentiated from bone marrow cells (BMCs) over 4 days in the presence of macrophage‐colony stimulating factor (M‐CSF) and RANKL at indicated concentrations of soluble FKN, and areas covered by tartrate‐resistant acid phosphatase‐positive (TRACP+) cells were measured. Data are presented as means ± SEM. (Right) Representative images of cells either untreated (upper panel) or treated (lower panel) with 10 nM soluble FKN. Scale bars, 300 μm. Figure S2. Cell surface expression of CX3CR1 on CD11b− CD115− cells 2 days after macrophage‐colony stimulating factor (M‐CSF) stimulation. BMCs were cultured 2 days in the presence of M‐CSF and then analyzed by flow cytometry. (Left) Representative data plot. (Right) CX3CR1 (upper panel) and RANK (lower panel) expression on CD11b− CD115− cells. Gray lines, isotype IgG; red lines, anti‐CX3CR1 mAb or anti‐receptor activator of NF‐κB (RANK) monoclonal antibody. Figure S3. Decreased cell surface expression of CX3CR1 on CD11bhigh CD115+ osteoclast precursors (OCPs) at 1 day after receptor activator of NF‐κB ligand (RANKL) stimulation. Bone marrow cells (BMCs) were cultured 2 days in the presence of macrophage‐colony stimulating factor (M‐CSF) followed by RANKL stimulation for 1 day and subsequent flow cytometry analysis. (Left) Representative data plot. (Right) CX3CR1 fraction gated for CD11bhigh CD115+. Gray and red lines: cells grown without and with immobilized fractalkine, respectively. Figure S4. Bone histomorphometry analysis osteoclast parameters at acute phase of receptor activator of NF‐κB ligand (RANKL)‐induced bone loss. Quantitative analysis of trabecular bone volume per tissue volume (BV/TV), osteoclast number per bone perimeter (OC number/Perim), and osteoclast area per bone perimeter (OC area/Perim) in femoral sections. Femurs were isolated from control PBS‐treated m [file JBM4-6-e10680-s001.pdf]

Supplemental Fig. S1

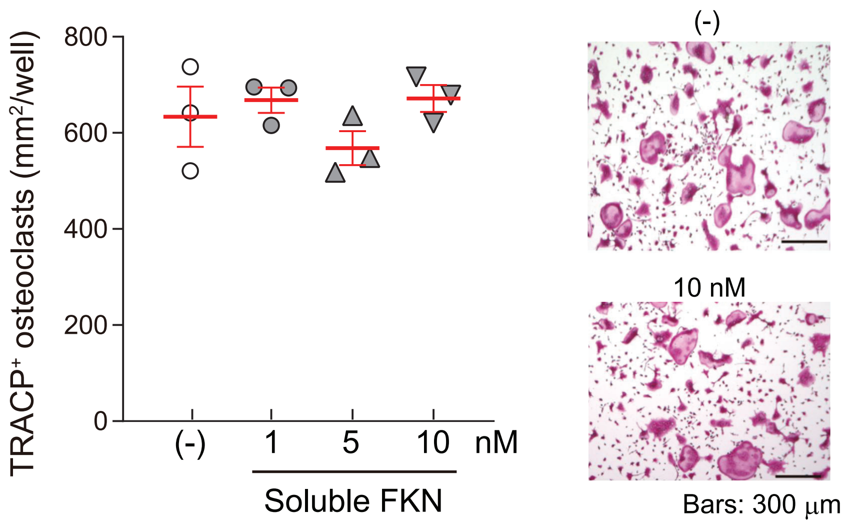

Supplemental Fig. S1. Effect of soluble FKN on RANKL-induced osteoclast formation in vitro. (Left) Osteoclasts were differentiated from BMCs over 4 days in the presence of M-CSF and RANKL at indicated concentrations of soluble FKN, and areas covered by TRACP^{+} cells were measured. Data are presented as means ± SEM. (Right) Representative images of cells either untreated (upper panel) or treated (lower panel) with 10 nM soluble FKN. Scale bars, 300 μm.

Supplemental Fig. S2

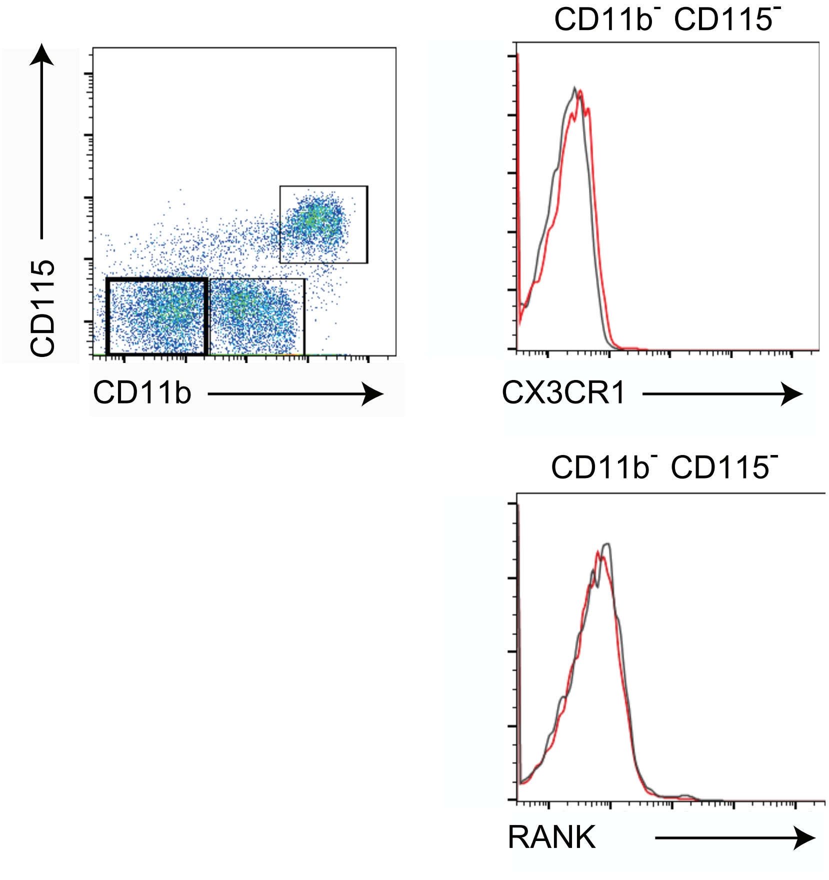

Supplemental Fig. S2. Cell surface expression of CX3CR1 on CD11b⁻ CD115⁻ cells 2 days after M-CSF stimulation. BMCs were cultured 2 days in the presence of M-CSF and then analyzed by flow cytometry. (Left) Representative data plot. (Right) CX3CR1 (upper panel) and RANK (lower panel) expression on CD11b⁻ CD115⁻ cells. Gray lines, isotype IgG; red lines, anti-CX3CR1 mAb or anti-RANK mAb.

Supplemental Fig. S3

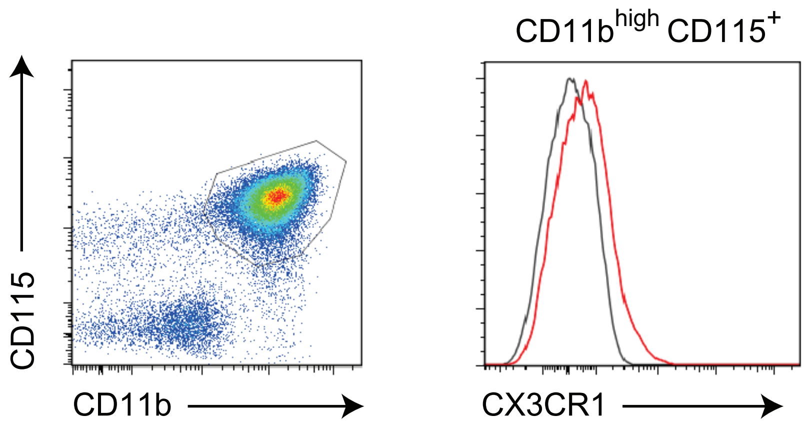

Supplemental Fig. S3. Decreased cell surface expression of CX3CR1 on CD11b^{high} CD115^{+} OCPs at 1 day after RANKL stimulation. BMCs were cultured 2 days in the presence of M-CSF followed by RANKL stimulation for 1 day and subsequent flow cytometry analysis. (Left) Representative data plot. (Right) CX3CR1 fraction gated for CD11b^{high} CD115^{+}. Gray and red lines: cells grown without and with immobilized FKN, respectively.

Supplemental Fig. S4

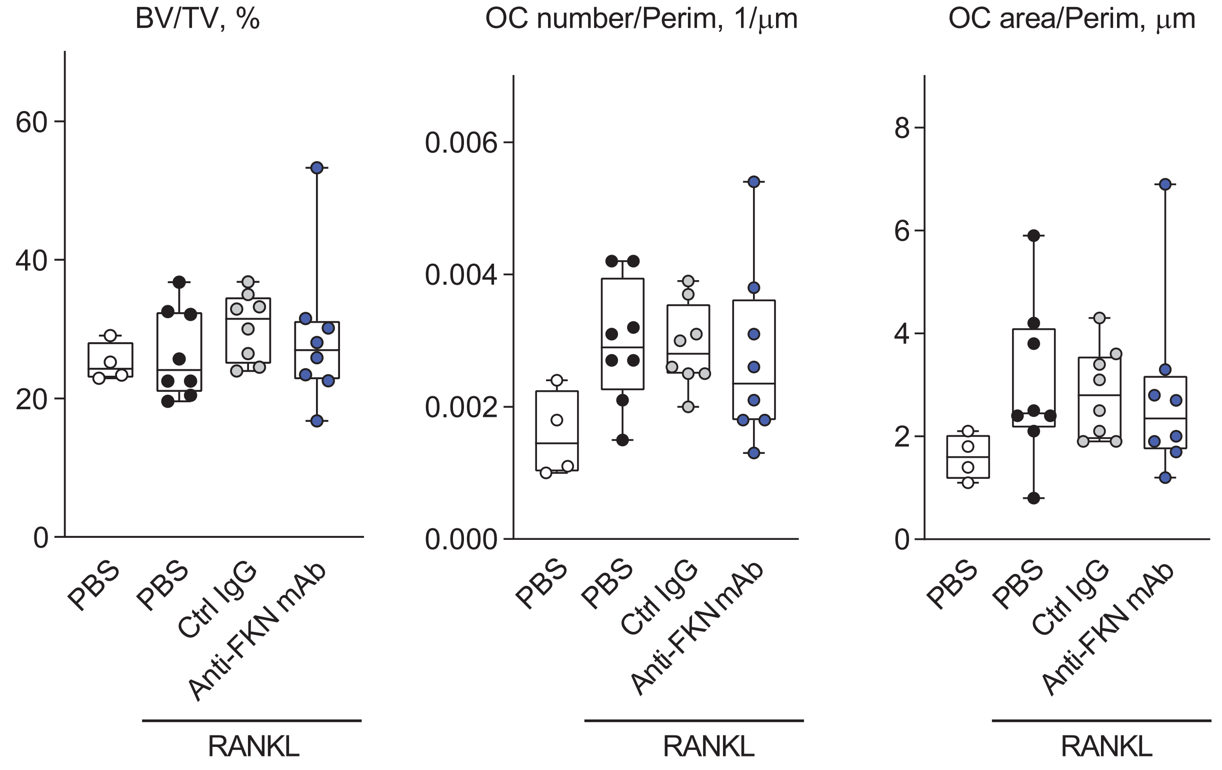

Supplemental Fig. S4. Bone histomorphometry analysis osteoclast parameters at the acute phase of RANKL-induced bone loss. Quantitative analysis of trabecular bone volume per tissue volume (BV/TV), osteoclast number per bone perimeter (OC number/Perim), and osteoclast area per bone perimeter (OC area/Perim) in femoral sections. Femurs were isolated from control PBS-treated mice (n=4) or RANKL-treated mice pretreated with PBS, control IgG, or anti-FKN mAb (clone 5H8-4) (n=8 per group). Box plots indicate the median, interquartile range, maximum and minimum.
